# Bidirectional regulation of bone formation by exogenous and osteosarcoma-derived Sema3A

**DOI:** 10.1038/s41598-018-25290-2

**Published:** 2018-05-02

**Authors:** Daniëlle de Ridder, Silvia Marino, Ryan T. Bishop, Nathalie Renema, Chantal Chenu, Dominique Heymann, Aymen I. Idris

**Affiliations:** 1 Department of Oncology and Metabolism, University of Sheffield, Medical School, Beech Hill Road, Sheffield, S10 2RX UK; 2 INSERM, UMR1238, Université de Nantes, Faculty of Medicine, 1 rue Gaston Veil, 4035 Nantes cedex 1, France; 3 INSERM, UMR 1232, Institut de Cancérologie de l’Ouest, Université de Nantes, Bd Jacques Monod, 44805 Saint-Herblain, France; 4 Department of Comparative Biomedical Sciences, Royal Veterinary College, London, NW1 0TU UK

## Abstract

Semaphorin 3A (Sema3A), a secreted member of the Semaphorin family, increases osteoblast differentiation, stimulates bone formation and enhances fracture healing. Here, we report a previously unknown role of Sema3A in the regulation of ectopic bone formation and osteolysis related to osteosarcoma. Human recombinant (exogenous) Sema3A promoted the expression of osteoblastic phenotype in a panel of human osteosarcoma cell lines and inhibited the ability of these cells to migrate and enhance osteoclastogenesis *in vitro*. *In vivo*, administration of exogenous Sema3A in mice after paratibial inoculation of KHOS cells increased bone volume in non-inoculated and tumour-bearing legs. In contrast, Sema3A overexpression reduced the ability of KHOS cells to cause ectopic bone formation in mice and to increase bone nodule formation by engaging DKK1/β-catenin signalling. Thus, Sema3A is of potential therapeutic efficacy in osteosarcoma. However, inhibition of bone formation associated with continuous exposure to Sema3A may limit its long-term usefulness as therapeutic agent.

## Introduction

Osteosarcoma is an aggressive form of skeletal cancer, typically with childhood and adolescent onsets^1–3^. Metastasis is a serious clinical manifestation of osteosarcoma^4,5^. However, skeletal complications such as osteolysis and rapid production of ectopic “woven” bone cause skeletal fractures and pain^4,5^. Thus, treatments aimed at reducing bone damage and halting metastasis would prove to be beneficial in terms of clinical outcomes and quality of life of osteosarcoma patients.

Semaphorins belong to a highly conserved family of secreted and membrane-associated signaling proteins^6,7^. Of the 8 different classes, only class 3 semaphorins (Sema3) are secreted^8–10^. Type A Sema3 (Sema3A) is synthesized as a precursor protein and proteolytically processed into two active secreted forms of 95 and 65 kilodaltons^8–10^. Secreted Sema3A is implicated in the migration of neuronal precursor cells and guided elongation and branching patterns of neurons^11–15^. Sema3A, and its co-receptors Neuropilin (Nrp)1 and 2^16–18^, are expressed by a variety of human cancer cells. Engagement of Sema3A with Nrp1 and 2 and other receptors has shown to both enhance and reduce tumour cell motility and invasion in various preclinical models of cancer^19–24^. Recent studies have reported that osteosarcoma tumours and cell lines overexpress both Nrp1 and Nrp2. Furthermore, Nrp2 expression is associated with poor prognosis and reduced overall survival in osteosarcoma patients^25–27^, and its knockdown in the human osteosarcoma cell line 143B inhibited tumour growth and reduced lung metastasis in mice^27^.

The Sema3A/Nrp axis plays a key role in the regulation of bone development and remodelling^28–30^. Sema3A, Nrp1/2 and Plexin-A1 are highly expressed in hypertrophic chondrocytes during embryonic development and in blood vessels and nerves at later stages of bone development^31^. Nrp1 and Nrp2 are expressed by osteoblasts and osteocytes, and mice deficient in Nrp2 exhibited bone loss due to enhanced osteoclast and reduced osteoblast numbers^32^. Recently, Hayashi and colleagues have reported that osteoblasts secrete Sema3A, and adult mice deficient in Sema3A had low peak bone mass mainly due to enhanced bone resorption and reduced osteoblast differentiation^29,32^. Furthermore, administration of Sema3A in mice enhanced osteoblast differentiation and bone formation, promoted fracture healing in osteoporotic rats and protected against bone loss due to oestrogen deficiency^29,31,33^.

In view of the fact that Sema3A is highly expressed in and secreted by cells of osteoblastic origin, and previous findings that implicated Nrp1 and Nrp2 receptors in osteosarcoma^26,27^, we examined the role of Sema3A in osteolysis and abnormal bone formation associated with osteosarcoma. In the present study, we report that human recombinant (exogenous) and osteosarcoma-derived Sema3A reduced the ability of osteosarcoma cells to enhance osteoclastogenesis *in vitro* and *in vivo* but it exerted differential effects on osteosarcoma associated osteoblast changes and ectopic bone formation *in vivo*.

## Results

### Recombinant Sema3A inhibits migratory, osteoblastic and osteolytic features of osteosarcoma cells

Previous studies have shown that Sema3A enhances osteoblast differentiation but its effects on the metastatic and osteolytic behaviour of the osteoblast-like osteosarcoma cells have not been investigated. With this in mind, we tested the effect of human recombinant Sema3A (exogenous) on the ability of various human osteosarcoma cell lines to grow, migrate, express osteoblast features and influence osteoclast formation *in vitro*. The concentration of Sema3A (300 ng/ml) used has been chosen on the basis of previous studies that have shown to stimulate osteoblast differentiation and inhibit osteoclast formation *in vitro*^29^. Exposure of osteoblast-like cells MC3T3, the more differentiated human osteosarcoma cells MG-63 and Saos-2 and the highly metastatic MNNG/HOS and KHOS cells to exogenous Sema3A (300 ng/ml) had no effect on their viability after 48 hours (Supplementary Figure S1). As shown in Table 1, exposure to exogenous Sema3A (300 ng/ml) reduced the directed migration of the osteoblast-like cell MC3T3 and the human osteosarcoma cell lines MG-63, MNNG/HOS (p < 0.01), KHOS and Saos-2 (p < 0.05). Exposure of the osteoblast-like cell MC3T3 and the more differentiated osteosarcoma cells MG-63 and Saos-2 to exogenous Sema3A (300 ng/ml) enhanced alkaline phosphatase activity (Table 1, p < 0.001, p < 0.01 and p < 0.05 respectively), without affecting cell viability (Supplementary Figure S1). Furthermore, M-CSF pre-treated mouse bone marrow derived osteoclast precursors and RAW 264.7 cells (pre-osteoclasts) with exogenous Sema3A (300 ng/ml) prior to addition of human osteosarcoma cell lines significantly inhibited osteoclast formation (Table 1 p < 0.01, p < 0.001), indicative of anti-osteoclast effects. These *in vitro* results suggest that treatment with Sema3A may be beneficial to reduce osteosarcoma growth and metastasis *in vivo*.

**Table 1:** Effect of exogenous Sema3A on the behaviour of human osteosarcoma cells *in vitro*.

	MC3T3	MG-63	Saos-2	MNNG/HOS	KHOS
Cell migration	−21 ± 2.3**	−29 ± 4.4**	−50 ± 9.3*	−18 ± 1.9**	−18 ± 3.1*
Alkaline phosphatase activity	+33 ± 1.5***	+79 ± 11.2**	+24 ± 6.3*	+3 ± 5.4	ND
OS-associated osteoclastogenesis	−23 ± 2.2**	−19 ± 0.7**	−48 ± 1.3***	−51 ± 2.0**	−48 ± 8.3**

Cancer cell migration, osteoblast differentiation and osteoclast formation were assessed as described under Materials and Methods.

Values are expressed as means ± SEM and are obtained from 3 different experiments. *p < 0.05, **p < 0.01, ***p < 0.001 from

vehicle treated cultures. OS denotes osteosarcoma and ND denotes non-detected.

### Exogenous Sema3A enhances bone volume

**Figure 1:**
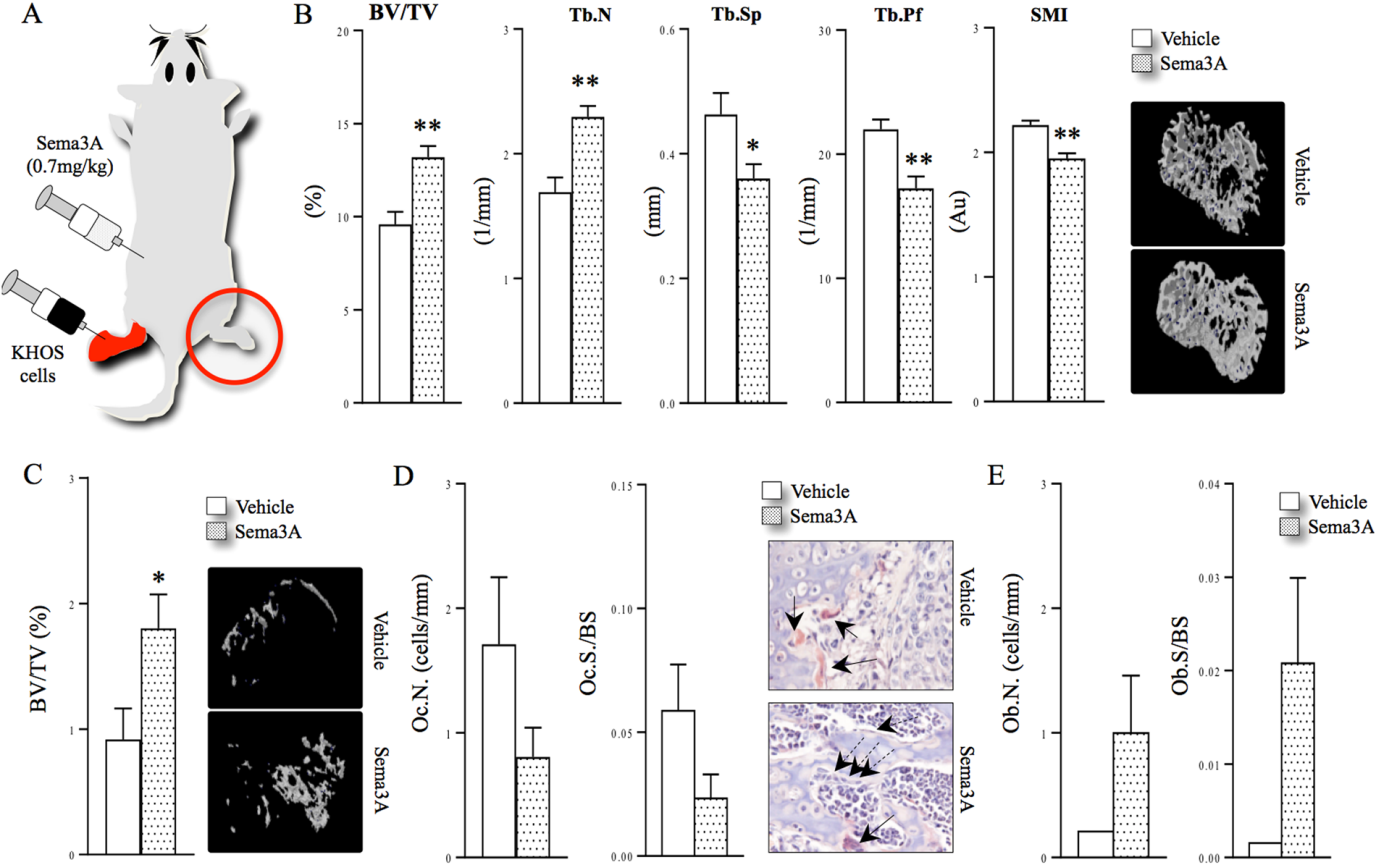
Exogenous Sema3A enhances bone volume *in vivo*. (**A**) Graphic representation of para-tibial injection of human KHOS cells treated with Sema3A (0.7 mg/kg) or vehicle (n = 7, 21 days) in 5 week old female Rj: NMRI nude mice. (**B**) Bone volume (BV/TV) and trabecular number (Tb.N), separation (Tb.Sp.), pattern factor (Tb.Pf) and structure model index (SMI) from non-inoculated tibia (solid circle) from the mice described in panel A. Representative photomicrographs of microCT scan of trabecular bone from the experiment described are shown in the right. (**C**) Bone volume (BV/TV) from tumour-bearing tibia from the mice described in panel A. Representative photomicrographs of microCT scan of trabecular bone from the experiment described are shown on the right. (**D**) *In vivo* osteoclast number (Oc.N, left) and osteoclast surface (Oc.S/BS, middle) from tibial metaphysis from mice from the experiment described in panel C. Representative photomicrographs of TRAcP positive osteoclasts indicated with solid arrows and osteoblasts indicated with dotted arrows from the experiment described are shown on the right. (**E**) *In vivo* osteoblast number (Ob.N, left) and active osteoid surface (Ob.S/BS, middle) from tibial metaphysis from mice from the experiment described in panel C-E. Data are mean ± SEM, n = 7 and n = 5 for histomorphometry *p < 0.05, **p < 0.01.

Sema3A enhances bone volume in mice^29,32^. In this study, we examined its effects on bone remodelling associated with osteosarcoma using the human KHOS mouse model. Human recombinant Sema3A (0.7 mg/kg/2-weekly) was administered in young mice 2 days after the local paratibial injection of parental human KHOS osteosarcoma cells (1.0 × 10^6^) and continued until day 21. Doses and treatment regime for Sema3A have been chosen on the basis of previous studies that have shown that Sema3A reduces ovariectomy-induced bone loss^29^. Analysis of bone architecture in the non-tumour-bearing leg confirmed that Sema3A (0.7 mg/kg/2-weekly) enhanced bone volume (% BV/TV, tibia 38% ± 6.1, femur 70% ± 13 p < 0.01) and trabecular number (% Tb.N, tibia 38% ± 5.1, femur 58% ± 9.5 p < 0.01), and connectivity (% Tb.Pf, tibia 22% ± 4.4, femur 32% ± 4.8 p < 0.01) and reduced trabecular separation (% Tb.Sp, tibia 22% ± 5.0, femur 27 ± 6.0% p < 0.05) in the control (Fig. 1 and Supplementary Figure S2). These results are in confirmation with previous findings and suggest that Sema3A may be of value in diseases involving the bone homeostasis such as osteoporosis. In the tumour-bearing tibia, administration of Sema3A (0.7 mg/kg/2-weekly) enhanced bone volume (Fig. 1C, % BV/TV, 96% ± 30, p < 0.05) and showed a trend towards, reduced osteoclast number (Fig. 1D, Oc.N/BS, left panel) and activity (Fig. 1D, Oc.S/BS, right panel) and increased osteoblast number (Fig. 1E Ob.N/BS, left panel) and active surface (Fig. 1E, Ob.S/BS, right panel). In keeping with these observations, we observed a trend towards reduced serum level of the bone resorption marker C-terminal telopeptide crosslinks (CTX) and enhanced level of the bone formation marker N-terminal propeptide of type 1 procollagen (P1NP) (Figure S2B). Administration of exogenous Sema3A had no effect on tumour volume (Figure S3A), but we observed a trend towards reduced number of micro-metastasis in the lungs (Figure S3B). The aforementioned observations imply that while Sema3A treatment may not be beneficial to treat osteosarcoma, it may be of value to prevent osteosarcoma-associated osteolysis.

### Osteosarcoma-derived Sema3A reduces KHOS cell growth, motility and invasion ***in vitro***

**Figure 2:**
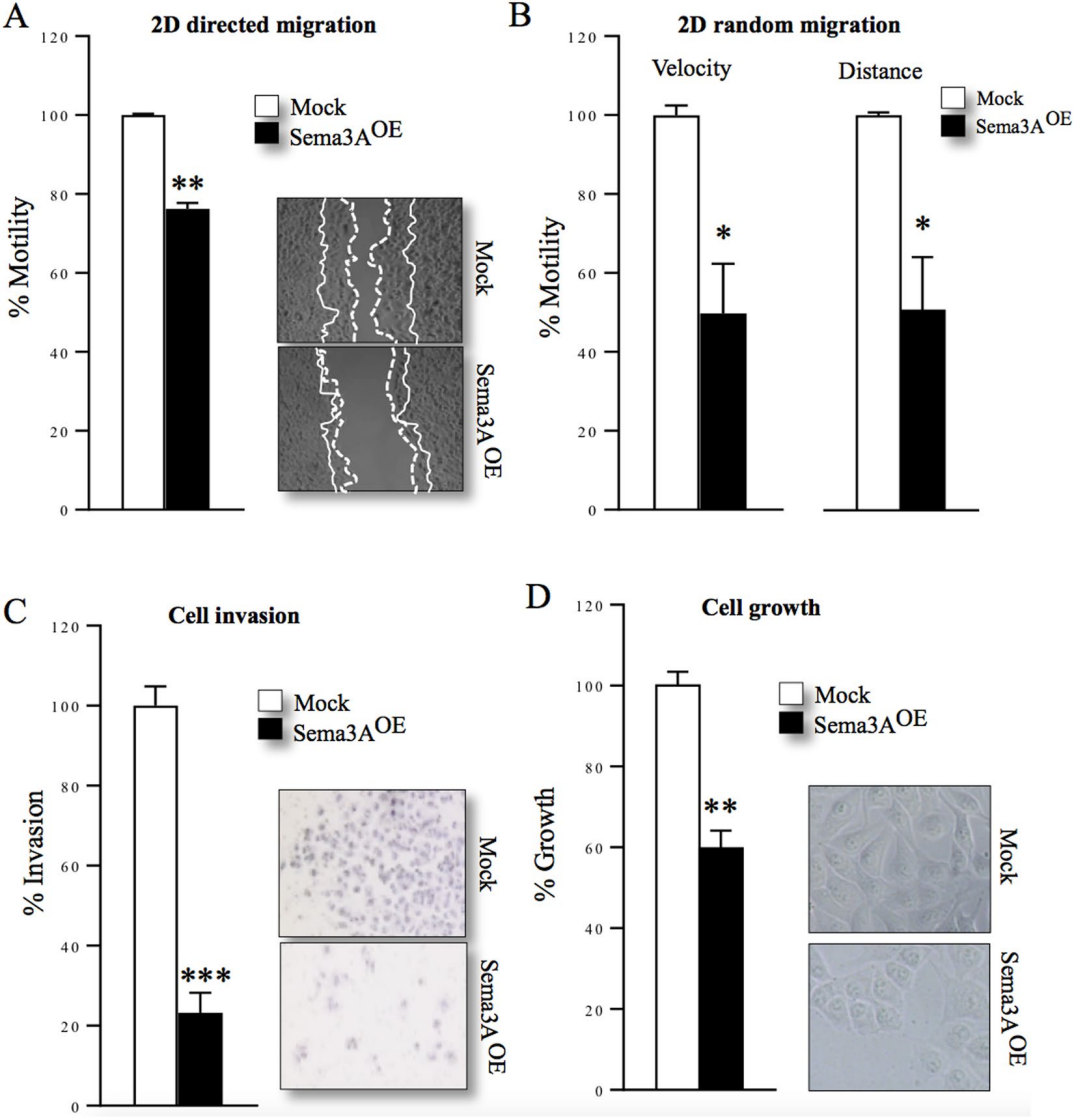
Osteosarcoma-derived Sema3A reduces motility, invasion and growth of KHOS cells. *In vitro* 2D directed (**A**) and random (**B**) cell migration of human KHOS cells overexpressing Sema3A (Sema3A^OE^) or mock control after 4 and 8 hours as assessed by wound healing and individual cell tracking, respectively. Representative photomicrographs from the 2D directed migration experiment described are shown in panel A, right. (**C**) *In vitro* cell invasion of human KHOS cells overexpressing Sema3A (Sema3A^OE^) or mock control after 72 hours as assessed by Transwell Chamber assay. Representative photomicrographs from the experiment described are shown in the right. (**D**) *In vitro* cell growth of human KHOS cells overexpressing Sema3A (Sema3A^OE^) or mock control in the absence of fetal calf serum after 48 hours as assessed by AlamarBlue assay. Representative photomicrographs from the experiment described are shown in the right. A line represents cell front at 0 hours, dotted line represents cell front at 4 hours. Data are mean ± SEM, n = 3 *p < 0.05, **p < 0.01 ***p < 0.001.

Previous studies have shown that primary osteoblasts express Sema3A, and osteosarcoma cells express its co-receptor Nrp1 and 2^29,31,34^. Here, we show that a panel of human and murine osteosarcoma cell lines express (Figure S4A) and secrete (Figure S4B) Sema3A. Interestingly, Sema3A expression was reduced in the metastatic osteosarcoma cells MNNG/HOS, POS-1, MOS-J and K7M2 when compared to the osteoblast-like cells Saos-2 (Figure S4A and B). This finding has led us to hypothesize that osteosarcoma-derived Sema3A is implicated in the behaviour of osteosarcoma cells in bone. To examine this hypothesis, we stably overexpressed Sema3A in the highly metastatic human KHOS cells (2.5 fold in cell lysate, Figure S4C,D and 8.5 fold in conditioned medium, S4E-F; p < 0.05), and showed Sema3A overexpression significantly reduced directed migration (Fig. 2A, 24% ± 1.5, p < 0.01) and random movement by up to 50% ± 13 within 4 hours (p < 0.05) as evidenced by reduced velocity and accumulated distance (Fig. 2B). Moreover, Sema3A overexpression in KHOS cells reduced cell invasion (Fig. 2C, 77% ± 5, p < 0.001,) after 72 hours. Sema3A overexpression only inhibited the growth of KHOS after 48 hours of continuous culture in absence of fetal calf serum (Fig. 2D, 40% ± 4.5, p < 0.01). This suggests that autocrine secretion of Sema3A by human KHOS cells suppresses their growth and motility *in vitro*.

### Osteosarcoma-derived Sema3A reduces ectopic bone formation

**Figure 3:**
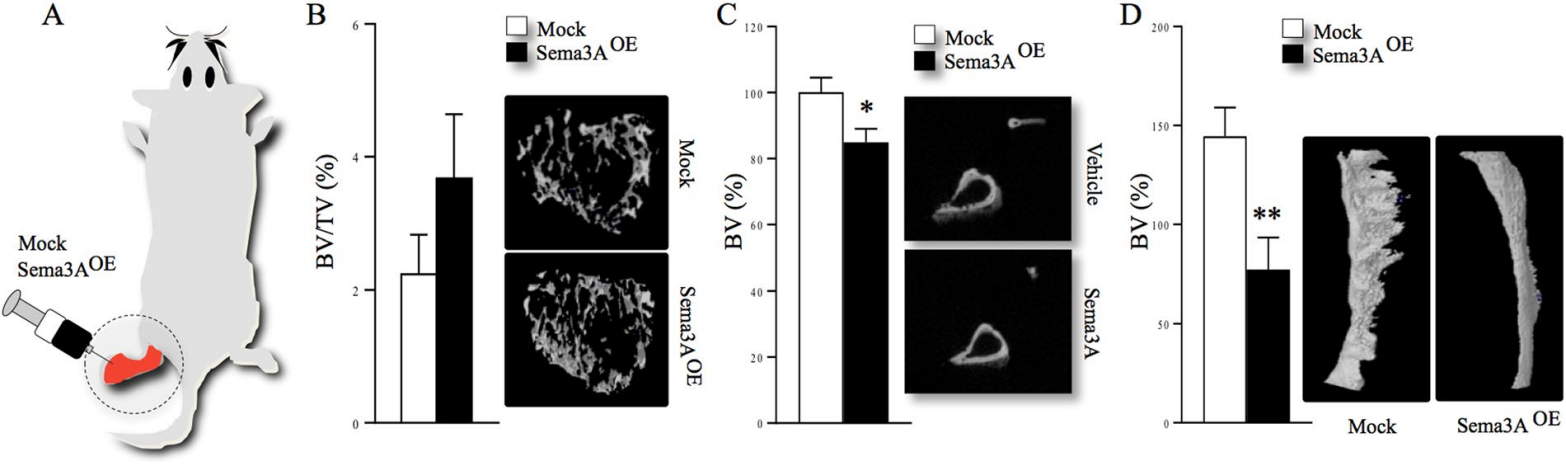
Osteosarcoma-derived Sema3A inhibits ectopic bone formation *in vivo*. (**A**) Graphic representation of para-tibial injection of human KHOS cells overexpressing Sema3A (Sema3A^OE^) or control (mock) (n = 10, 16 days) in 5 week old female Rj: NMRI nude mice. (**B**) Bone volume (BV/TV, %) in the tumour-bearing tibia (dashed circle) from the experiments described in panel A. Representative photomicrographs of microCT scan of trabecular bone from the experiment described are shown on the right. Ectopic bone volume (BV %) in the tumour-bearing tibia (**C**) and fibula (**D**) from the experiments described in panels A-B. Representative photomicrographs of microCT scans of fibular ectopic bone from the experiment described are shown on the right. Data are mean ± SEM, n = 10 *p < 0.05, **p < 0.01.

Subsequently, we examined the ability of KHOS cells overexpressing Sema3A to cause osteolysis in mice. As shown in Fig. 3, para-tibial inoculation of human KHOS osteosarcoma cells caused severe bone loss with 75% of trabecular bone volume completely lost within 16 days (Fig. 3A,B). Nonetheless, mice inoculated with human KHOS osteosarcoma cells overexpressing Sema3A exhibited a trend towards more bone volume indicative of a modest osteoprotective effect (Fig. 3A,B). In view of the fact that osteosarcoma cells induce the formation of ectopic bone in humans^35^, we carried out detailed microCT analysis of bone indexes in the fibula and proximal tibia of mice. This analysis revealed that mice inoculated with Sema3A overexpressing KHOS cells exhibited reduced ectopic bone volume in both fibula and tibia (Fig. 3C,D, fibula 67% ± 16 p < 0.05; tibia 15% ± 4 p < 0.01) when compared to control mice. Representative photomicrographs of ectopic bone formation from the experiment described are shown in (Fig. 3C and D, right). In contrast, administration of human recombinant Sema3A (0.7 mg/kg/2-weekly) had no effect on ectopic bone formation in mice (data not shown). Of note, we observed no differences in tumour growth in mice inoculated with mock or Sema3A overexpressing KHOS cells (Figure S3C). Together, these results imply that tumour-derived Sema3A reduces the ability of osteosarcoma cells to cause ectopic bone formation without affecting their growth.

### Differential effects of osteosarcoma-derived Sema3A on osteoblasts

**Figure 4:**
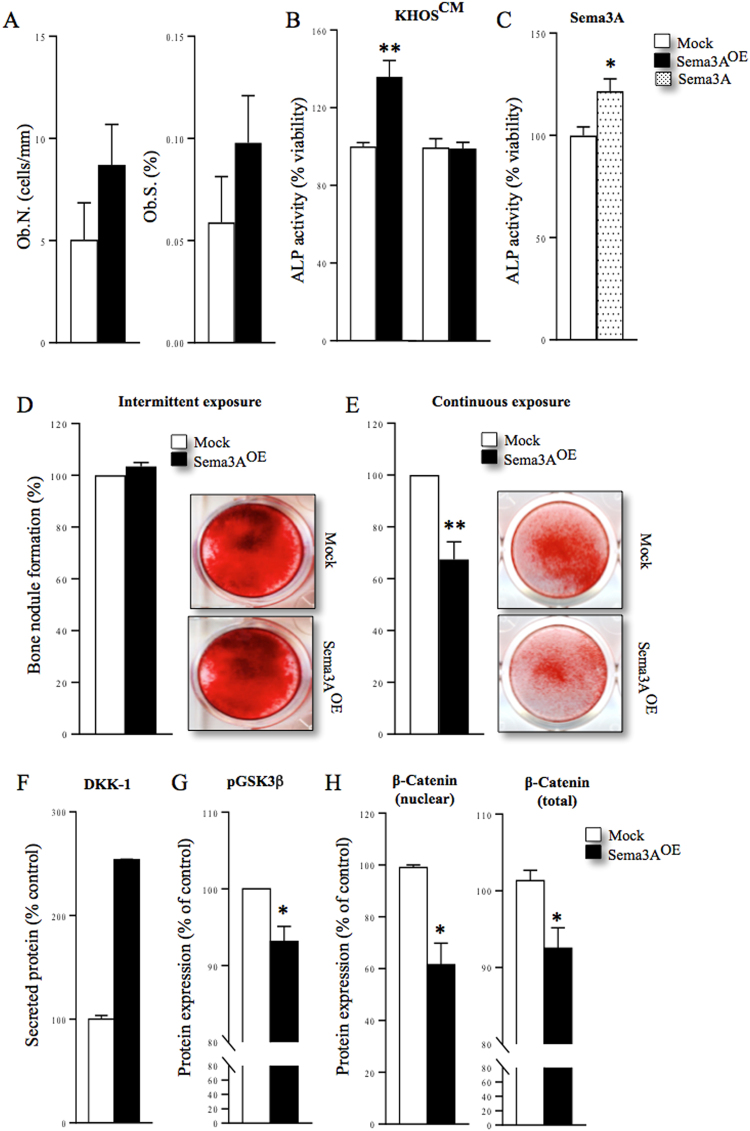
Bidirectional regulation of osteoblast differentiation by Sema3A. (**A**) *In vivo* osteoblast number (Ob.N) from tibial metaphysis from the mice of the experiment described in Fig. 3. (**B**,**C**) *In vitro* differentiation of Saos-2 after intermittent (**B**, left) and continuous (**B**, right) exposure to conditioned medium from human KHOS cells overexpressing Sema3A (Sema3A^OE^) or Sema3A (300 ng/ml, **C**) as assessed by alkaline phosphatase assay. *In vitro* bone nodule formation in Saos-2 after intermittent (**D**) and continuous (**E**) exposure to conditioned medium from human KHOS cells overexpressing Sema3A (Sema3A^OE^) or their respective controls as assessed by Alazarin Red. Representative photomicrographs of bone nodule formation from the experiments described are shown in the right. (**F**) Levels of tumour derived osteoblast inhibitory factor, DKK1 in conditioned medium from human KHOS cells overexpressing Sema3A (Sema3A^OE^) or their respective control measured by the Proteome Profiler Human XL Cytokine Array Kit. (**G**) Differential expression of p-GSK3β after 15 minutes in the osteoblast-like MC3T3 cells exposed to conditioned medium from human KHOS cells overexpressing Sema3A (KHOS^OE^) or their respective control (mock). (**H**) Differential expression of nuclear β-catenin (left) after 45 minutes and total β-catenin (right) after 24 hours in the osteoblast-like MC3T3 cells exposed to conditioned medium from human KHOS cells overexpressing Sema3A (KHOS^OE^) or their respective control (mock). Data are mean ± SEM, n = 3 *p < 0.05, **p < 0.01.

To explore the mechanism responsible for the bidirectional effects of exogenous and tumour-derived Sema3A on the skeleton, we tested their effects on bone cell growth, differentiation and signalling. Histomorphometrical analysis of bone from mice injected with KHOS cells overexpressing Sema3A revealed a trend towards increased osteoblast number (Fig. 4A, Ob.N/BS, 73% ± 39 left panel) and active osteoblast surface (Fig. 4A, Ob.S/BS, 66% ± 39 right panel). *In vitro*, intermittent exposure to conditioned medium from KHOS overexpressing Sema3A (Fig. 4B) or Sema3A (Fig. 4C, 300 ng/ml) enhanced alkaline phosphatase activity in Saos-2 cultures after 48 hours (p < 0.01), whereas continuous exposure to conditioned medium from KHOS overexpressing Sema3A (20%, v/v) had no effects (Fig. 4B, right). Continuous exposure to conditioned medium from KHOS overexpressing Sema3A significantly reduced the ability of Saos-2 to form bone nodules *in vitro* by up to 32 ± 6.8% (Fig. 4E, p < 0.01) compared to intermittent treatment (Fig. 4D, 3.4% ± 1.6). Representative photomicrographs of bone nodule formation from the experiments described are shown in Fig. 4 (panels D and E). It is important to note that neither exogenous nor tumour-derived Sema3A had an effect on the viability or growth of primary calvarial osteoblasts and the osteoblast-like cells MC3T3 and Saos-2 after up to 12 (Saos-2), 25 and 28 (MC3T3 and calvarial osteoblasts) days (Figure S5). These results together indicate that sustained exposure to tumour-derived Sema3A reduces the ability of osteosarcoma cells to form bone nodule without affecting cell viability.

### Osteosarcoma-derived Sema3A disrupts DKK1/β-catenin signalling

Canonical Wnt signaling regulates osteoblast differentiation and bone formation, and previous studies have shown that Sema3A activates β-catenin in bone cells^29^. Here, we show that Sema3A overexpression in KHOS cells significantly increased the level of DKK1 in conditioned medium as measured by Proteome Profiler Human XL Cytokine Array Kit (Fig. 4F). Consistently, exposure of osteoblast-like cells MC3T3 to conditioned medium from KHOS overexpressing Sema3A reduced GSK3β phosphorylation within 15 minutes, transient nuclear β-catenin within 45 minutes and β-catenin expression after 24 hours (Fig. 4G), indicative of inhibition of Wnt signaling. In contrast, exogenous Sema3A had no effect on either GSK3β or β-Catenin expression (data not shown). Together, these results implicate the autocrine DKK1/GSK3β/β-catenin signaling in the regulation of osteoblast differentiation by osteosarcoma-derived Sema3A.

### Osteosarcoma-derived Sema3A inhibits KHOS-induced osteoclastogenesis

**Figure 5:**
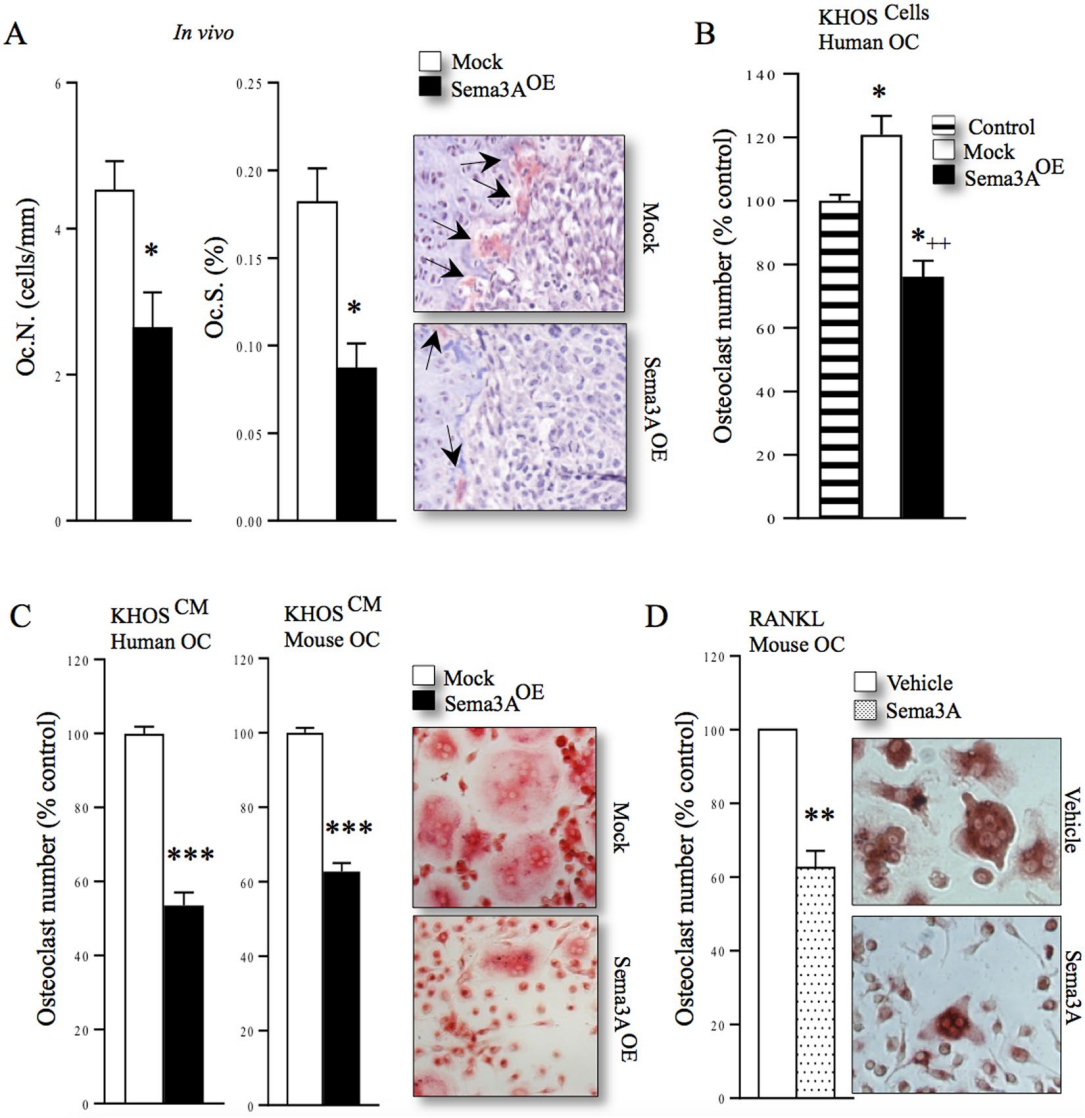
Osteosarcoma-derived Sema3A reduces osteotropic activity of KHOS cells. (**A**) *In vivo* osteoclast number (Oc.N, left) and osteoclast surface (Oc.S, middle) from tibial metaphysis from mice from the experiment described in Fig. 3. Representative photomicrographs of TRAcP positive osteoclasts indicated with solid arrows from the experiment described are shown on the right. (**B**) *In vitro* human osteoclastogenesis in RANKL-stimulated human peripheral blood monocytes co-cultured with human KHOS cells overexpressing Sema3A (Sema3A^OE^) or mock control as assessed by TRAcP staining. (**C**) *In vitro* mouse and human osteoclastogenesis in RANKL-stimulated human peripheral blood monocytes (left) and mouse bone marrow cells (middle) exposed to conditioned medium from human KHOS cells overexpressing Sema3A (Sema3A^OE^) or mock control. Representative photomicrographs of human osteoclasts from the experiment described are shown on the right. (**D**) *In vitro* osteoclastogenesis of murine M-CSF- and RANKL-stimulated bone marrow cultures in the presence and absence of Sema3A (300 ng/ml). Representative photomicrographs of mouse osteoclasts from the experiment described are shown in the right. Data are mean ± SEM, n = 3 *p < 0.05, **p < 0.01 ***p < 0.001, ^++^p < 0.01 of mock.

Previous studies have reported that Sema3A inhibits osteoclast formation and protects against bone loss^29,32^. Here, we find that mice injected with human KHOS osteosarcoma cells overexpressing Sema3A had reduced osteoclast numbers (Fig. 5A, Oc.N/BS) and suppressed osteoclast activity (Fig. 5A, Oc.S/BS). Additionally, we examined the effects of overexpression of Sema3A on the ability of KHOS to influence osteoclast formation *in vitro*. As shown in Fig. 5 (panel B), human KHOS cells significantly enhanced RANKL stimulated osteoclast formation in cultures of human peripheral blood monocytes, and this effect was significantly reduced in osteoclast cultures exposed to human KHOS cells overexpressing Sema3A (Fig. 5B, 45% ± 5, p < 0.01). Further *in vitro* experiments with conditioned medium from KHOS cells confirmed these effects and showed a significant reduction in osteoclast numbers in human peripheral blood monocytes and mouse bone marrow cultures exposed to conditioned medium (20%, v/v) from human KHOS cells overexpressing Sema3A (Fig. 5C, left 46% ± 3.5 and right: 37% ± 2.3, p < 0.001) when compared to control cultures. Similarly, we observed a 37% reduction in osteoclast numbers in RANKL-stimulated mouse bone marrow cultures treated with exogenous Sema3A (300 ng/ml, Fig. 4D).

## Discussion

Previous studies have shown that Sema3A is secreted by osteoblasts, and its co-receptors Nrp1 and 2 have been detected in human osteosarcoma cells^25–27,29,32^. Together, these findings have led us to hypothesize that Sema3A plays a role in the regulation of osteosarcoma associated bone remodelling. In support of this hypothesis, we show that the human recombinant Sema3A reduced the ability of human osteosarcoma cells to influence bone activity and its administration in mice enhanced bone volume in the absence and presence of KHOS cells in the bone microenvironment. These effects were indicative of the osteoprotective effects of Sema3A and were consistent with previous studies that have shown that Sema3A promoted fracture healing in osteoporotic rats, enhanced bone volume in mice during growth and after oestrogen deficiency^29,36^. Together with previous studies, our results indicate the importance of Sema3A in bone remodelling by suppression of bone resorption and enhancing bone formation. Further research is required but the coupled effect could make Sema3A an ideal treatment for osteolytic bone diseases such as osteoporosis^30,37^.

Ectopic bone formation is a key feature of osteosarcoma^35^. A surprising finding of the present study was that osteosarcoma-derived Sema3A inhibited the formation of new ectopic bone in mice inoculated with KHOS cells, contrasting with the osteoanabolic effects of Sema3A in mice reported in previous studies^29^. Our interpretation of these differences is that exogenous Sema3A and overexpression of Sema3A in KHOS cells exerts differential effects on the ability of osteosarcoma cells to enhance bone formation. Evidence to support this hypothesis comes from the experiments that showed that continuous exposure of osteoblast-like cells to conditioned medium from KHOS cells overexpressing Sema3A reduced their ability to form bone nodules *in vitro*. Similarly, continuous exposure of osteoblast-like cells to conditioned medium from KHOS cells overexpressing Sema3A also reduced alkaline phosphatase activity in cultures of Saos-2, indicative of an inhibitory effect on osteoblast maturation and differentiation. We excluded the possibility that exposure to conditioned medium from KHOS cells overexpressing Sema3A affects the viability of osteoblast-like cells in these cultures since we have not observed any changes in osteoblast number *in vitro* or *in vivo*.

Osteosarcoma cells influence osteoblast differentiation and bone formation by secretion of various mediators^38,39^. Although the signaling mechanisms by which Sema3A affects bone cell differentiation and function are yet to be fully characterised, we present evidence to suggest that DKK1/β-catenin signalling may be one mechanism by which Sema3A influences osteosarcoma-associated ectopic bone formation. In support of this, we show that overexpression of Sema3A in KHOS cells enhanced the production of the Wnt signaling and osteoblast inhibitor, DKK1 and conditioned medium from KHOS cells overexpressing Sema3A reduced β-catenin activation and expression in osteoblasts. These findings were consistent with the inhibitory effects of DKK1 in osteoblasts and in broad agreement with previous studies that have reported that Sema3A affects β-catenin activation in osteoblasts^29,40^. Whilst we can not exclude the contribution of various local and systemic factors implicated in the regulation of osteosarcoma cell – osteoblast crosstalk, the relative increase in secreted DKK1 by KHOS cells overexpressing Sema3A may provide a plausible mechanism for the differential effects of exogenous and tumour-derived Sema3A on ectopic bone formation in the model described.

Osteosarcoma cells promote osteolysis by enhancing osteoclast formation^41^. Previous studies have shown that Sema3A reduces osteoclast formation and protects against ovariectomy-induced bone loss in mice^29^. Our current data expand on these observations and demonstrate that both exogenous and tumour-derived Sema3A inhibited osteosarcoma-induced osteoclast formation and reduced osteosarcoma- and RANKL-induced osteoclast formation *in vitro*, indicative of a direct action on osteoclasts and their precursors. Similar osteoclast inhibition was observed in mice inoculated with Sema3A overexpressing cells, while a trend towards reduced osteoclast number was observed following administration of exogenous Sema3A in mice. These differences might be due to stronger osteoclast inhibition by tumour-derived Sema3A as opposed to that obtained by intermittent administration of exogenous Sema3A.

In spite of osteoclast inhibition and the significant reduction in osteosarcoma-associated bone remodelling by Sema3A, we have failed to detect a significant reduction in tumour growth in the KHOS model described. These findings differ from previous studies that have shown that Sema3A inhibits tumour growth in other cancer models^23,42,43^. We cannot readily explain these differences except to note that previous studies were performed in models using cell lines that are sensitive to Sema3A treatment due to high expression of the Sema3A co-receptors Nrp1 and 2^27^. We have consistently showed that Sema3A reduced the motility of a panel of osteosarcoma cell lines including KHOS, consistent with the anti-migratory action of osteosarcoma-derived Sema3A and the action of Sema3A previously reported in both neuronal and cancer cells^11–15,19–24^. Since Sema3A is known to selectively bind to its co-receptor Nrp1, further studies were conducted to determine if the anti-migratory effects of osteosarcoma derived Sema3A in our models were truly mediated by Nrp1. These experiments showed that the anti-migratory effect associated with osteosarcoma-derived Sema3A in KHOS cells was partially reversed by the Nrp1 antibody (Figure S6), indicating the involvement of other components of Semaphorin/Nrp signaling, in particular Nrp2 and vascular endothelial growth factor (VEGF)^28–30^. However, further studies are warranted and ongoing.

In conclusion, Sema3A plays a role in the osteoanabolic and metastatic properties of osteosarcoma cells and it inhibits osteosarcoma cell ability to stimulate osteoclastogenesis. Moreover, Sema3A enhances bone volume in mice inoculated with human osteosarcoma cells. Although these findings indicate that Sema3A may have a potential role in protecting the skeleton from osteosarcoma-associated osteolysis, our *in vivo* studies were restricted to the human KHOS model of immuno-deficient mice. Therefore, further studies will be required to determine if Sema3A is effective in reducing the development and progression of osteosarcoma in other models. Furthermore, our present results - when combined with published work^29,32^ - suggest that Sema3A has overlapping roles in regulating osteolysis, but it exerts differential effects on osteoblast differentiation and osteosarcoma associated ectopic bone formation. Thus, the potential use of Sema3A for the treatment of bone loss needs to be carefully tested so that any treatment regime would take into account the possibility of inhibition of bone formation that may limit Sema3A long-term usefulness as a bone sparing drug.

## Materials and Methods

### Reagents

The recombinant human Semaphorin 3A was purchased from R&D systems (Abingdon, UK). We used the human KHOS, MNNG/HOS, Saos-2 and MG-63, and mouse POS-1, MOS-J and K7M2 osteosarcoma cells (ATCC, Manassas, VA, USA). The mouse MC3T3 cells were used as a representative of osteoblasts and RAW 264.7 as macrophage-like, pre-osteoclast cells (ATCC, Manassas, VA, USA). Tissue culture medium (DMEM and alpha-MEM) was obtained from Gibco, Thermofisher (Leicestershire, UK). All primary antibodies were purchased from Cell Signalling Biotechnology (MA, USA) except rabbit anti-Semaphorin3A was purchased from Abcam (Abingdon, UK) and rabbit anti-actin was obtained from Sigma-Aldrich (Dorset, UK). Mouse macrophage colony stimulating factor (M-CSF) was obtained from R&D Systems (Abingdon, UK) and receptor activator of NFκB ligand (RANKL) was a gift from Patrick Mollat (Galapagos SASU, France), and was prepared as previously described^44^. Overexpression of Sema3A was achieved by use of control (sc-437282) and Sema3A lentiviral activation particles (sc-400716-LAC) Santa-Cruz (Heidelberg, Germany).

### Animal experiments

All experimental protocols were approved by the French ministry of Agriculture and were realized in accordance with the institutional guidelines of the regional ethical committee (Agreement number 1280.01, CREEA Pays de la Loire, France) and under supervision of authorized investigators. The effects of Sema3A overexpression and exogenous Sema3A in KHOS osteosarcoma cells on primary tumour growth and bone histomorphometry were studied by intramuscular para-tibial injection. Briefly, 34 female Rj: NMRI nude mice four-week old were purchased from Janvier Breedings (Le Genest Saint Isle, France) and allowed to acclimatize for a week after arrival. For the osteosarcoma-derived Sema3A experiment animals were divided into two groups of 10 mice and primitive osteosarcoma was induced by paratibial injection of 1.5 × 10^6^ human KHOS osteosarcoma cells or KHOS Sema3A overexpressing osteosarcoma cells. Weight and tumour volume was measured three times weekly. All mice were sacrificed 16 days after injection. The effects of Sema3A treatment on primary tumour growth and bone histomorphometry were studied using the KHOS model described. Animals were divided into two groups of 7 mice and primitive osteosarcoma was induced by intramuscular para-tibial injection of 1.0 × 10^6^ human KHOS osteosarcoma cells. Mice were given a biweekly injection of vehicle or 0.7 mg/kg Sema3A. All mice were sacrificed 21 days after injection. Healthy and tumour bearing legs were harvested for microCT (Brucker, Belgium) analysis. Lungs and tibial metaphysis were collected for immunohistochemistry, histomorphometry and metastasis analysis.

### Micro-computed tomography

Ectopic bone formation (tibia and fibula) and trabecular bone parameters (tibia and femur) were measured using microCT analysis (Skyscan 1076 Brucker, Belgium) set at 50 kV and 200 mA. Skyscan NRecon program was used to reconstruct the images and then analysed using Skyscan CTAn (Brucker, Belgium)^45^.

### Bone histomorphometry

Bone histomorphometry was performed on the proximal tibial metaphysis from mice as previously described^46^. In short, bones were fixed, sectioned using a Leica microtome (Solms, Germany) and tissue sections were stained with tartrate-resistant acid phosphatase (TRAcP) and haematoxylin and eosin (H&E). Histomorphometry was performed on trabecular bone distal from the growth plate (0.1 and 1 mm distal to the growth plate). Bone histomorphometrical analysis was performed as previously described^46^. Only TRAcP positive multi-nucleated cells that were in contact with the bone were counted as osteoclasts and measured for osteoclast surface. Osteoblasts were identified by their morphology and only counted if they were in contact with the bone.

### Biochemical Markers of Bone Turnover

Serum P1NP (a marker of bone formation) and CTX (a marker of bone resorption) were measured using mouse/rat competitive enzyme immunoassay kits (IDS; Boldon, UK), according to the manufacturer’s instructions.

### Assessment of cell viability

The AlamarBlue assay was used to measure the viability of osteosarcoma cells^47^. Vehicle or exogenous Sema3A (300 ng/ml) was added to the osteoblast like MC3T3 and osteosarcoma cells for the desired period. Cells were incubated in AlamarBlue reagent (DAL 1100 Thermofisher) (10%, vol/vol) for 3 hours and fluorescence was measured (excitation, 530 nm, emission 590 nm) using a SpectraMax® M5 microplate reader.

### Assessment of cell migration

Osteosarcoma cells KHOS, MNNG/HOS, MG-63 and Saos-2 were seeded in 24 well plates (0.15 × 10^6^) and osteoblast MC3T3-E1 at (0.1 × 10^6^). Once the cells reached confluency, the monolayer was wounded with a fine pipette tip and cells were treated with vehicle or exogenous Sema3A (300 ng/ml) for 16 hours. Migration of cells was visualized on a Leica AF6000 Time Lapse microscope. Percentage of wound closure was determined using TScratch. For random migration, KHOS osteosarcoma cells were plated in 24 well plates (1 × 10^3^ cells/well). Cells were treated with vehicle or Sema3A (300 ng/ml). Migration was monitored for 8 hours with a Leica AF6000 Time Lapse microscope. Accumulated distance (total track length) and velocity were measured using the Chemotaxis and Migration tool in ImageJ.

### Assessment of cell invasion

Cancer cell invasion was measured using Corning™ transwell inserts coated with Matrigel 1.5 mg/ml (Corning, UK). KHOS osteosarcoma cell suspensions (5 × 10^3^) were prepared in serum free medium containing vehicle or exogenous Sema3A (300 ng/ml) were pipetted onto the Matrigel. Outer wells were filled with standard DMEM. After 72 hour incubation under standard culture conditions, the cells were fixed and stained with haematoxylin and eosin.

### Assessment of osteoblast differentiation

Primary osteoblasts were isolated from the calvarial bones of 2-day-old mice as previously described^48^. Primary calvarial osteoblasts or the MC3T3 were seeded into 96-well plates at 7 × 10^3^ cells and 5 × 10^3^ cells per well respectively in standard alpha-MEM. KHOS, MNNG/HOS, Saos-2 and MG-63 osteosarcoma cells were seeded into 96-well plates at 4 × 10^3^ cells per well in standard-DMEM. After 24 hours cells were treated with vehicle or exogenous Sema3A (300 ng/ml) in serum free medium with the exception of primary osteoblasts in standard alpha-MEM. Cell viability and differentiation were determined by AlamarBlue assay and alkaline phosphatase (Alk Phos) assay^49^. For mineralization experiments, Saos-2 were treated with conditioned medium 20% (v/v) in osteogenic medium (50 μg/ml Ascorbic Acid, 10 nM Dexamethasone and 2 mM β–Glycerophosphate) every 48 hours for 9 days. In case of intermediate exposure, Saos-2 were exposed to conditioned medium 20% (v/v) in osteogenic medium for 6 hours out of the 48 hour cycle.

### Assessment of osteoclast formation

Osteoclast formation was studied by RANKL and M-CSF stimulated bone marrow cultures and M-CSF generated macrophage cultures, M-CSF and RANKL stimulated human primary CD14^+^ monocytes and RAW 264.7 cells. Bone marrow (BM) cells were flushed from the long bones of 3–5 week old mice as previously described^48^. Mouse M-CSF-dependent osteoclast precursor cells were generated as previously described^50^ and were plated into tissue culture plates (96 well plates, 15 × 10^3^ cells/well) in standard alpha-MEM supplemented with M-CSF (25 ng/ml) and RANKL (100 ng/ml) after 6 hours MNNG/HOS or Saos-2 osteosarcoma cells (500 cells/well) and vehicle or Sema3A (300 ng/ml) were added. Bone marrow cultures were plated at 45 × 10^3^ cells/well in standard alpha-MEM supplemented with M-CSF (25 ng/ml) and RANKL (100 ng/ml) for 24 h prior to addition of Sema3A (300 ng/ml) or vehicle and KHOS osteosarcoma cells 250 cells/well or their conditioned medium (20% v/v) prepared as previously described in^50^. Human CD14^+^ monocytes were isolated using a Ficoll gradient, selected with CD14^+^ MACS beads (Miltenyi Biotech, Germany) plated at 45 × 10^3^ and in standard alpha-MEM supplemented with M-CSF (20 ng/ml) for 48 hours prior to the addition of RANKL (100 ng/ml) and Sema3A (300 ng/ml) or vehicle and KHOS osteosarcoma cells 100 cells/well or their conditioned medium (20% v/v). RAW264.7 cells were plated at 400 cells/well and after 24 hours supplemented with RANKL (50 ng/ml), vehicle or Sema3A (300 ng/ml) and MG-63 or MC3T3 250cells/well. Cultures were terminated by fixation in 4% paraformaldehyde, and stained for Tartrate-Resistant Acid Phosphatase (TRAcP)^51^.

### Measurement of protein expression

Western blot analysis was used to detect protein expression. KHOS osteosarcoma cells were grown to 80% confluency until lysis. MC3T3 cells were incubated with vehicle, Sema3A (300 ng/ml) or conditioned medium 20% (v/v) from mock or Sema3A overexpressing KHOS cells. Cells were exposed to their conditions for the desired period of time and then gently scraped in standard lysis buffer (0.1% (w/v) SDS, 0.5% (w/v) sodium deoxycholate, 1% Triton X-100, 1 μM EDTA, 2% (v/v) protease inhibitor cocktail, 10 µM of sodium fluoride and 2% (v/v) phosphatase inhibitor cocktail). Protein concentration was determined using BCA assay (Pierce, USA). Total protein (50–80 μg) was resolved by SDS-PAGE (BioRAD, UK) and immunoblotted with antibodies according to manufacturer’s instructions. Native and phosphorylated proteins were detected using rabbit monoclonal antibodies β-catenin, GSK3β and pGSK3β (Ser9) (1:1000 dilution, Cell Signalling Technology, USA). Rabbit anti-Semaphorin3A and anti-Nrp1 monoclonal antibodies were purchased from Abcam (UK). Immuno-complexes were visualised by a chemiluminescence detection kit (BioRAD) using horseradish peroxidase-conjugated secondary antibody (Jackson laboratories), and visualised using chemiluminescence on a Chemidoc Imaging system (BioRAD). Bands were quantified using Image Lab Software (BioRAD) and level of actin (Sigma-Aldrich, UK) was used for normalization.

### Protein expression in conditioned medium

Sema3A expression in conditioned medium was measured by western blot. Cells were incubated for 16 hours with serum free medium. Conditioned medium was collected and concentrated using Pierce Protein Concentrators, 9 K MWCO (Thermofisher). Protein concentration was measured and protein was resolved by SDS page. Sema3A was detected using anti-Sema3A antibody and Sema3A protein was quantified using standard recombinant Sema3A (R&D systems, UK). DKK-1 expression in the conditioned medium was measured with the Proteome Profiler Human XL Cytokine Array Kit by R&D systems.

### Measurement of mRNA expression

Gene expression was detected using quantitative PCR (qPCR). Briefly, samples were lysed using Nucleospin RNA isolation kit (Macherey-Nagel) and quantified using a nanodrop (Thermo Scientific) according to manufacturer’s instructions. Complementary DNA (cDNA) was generated using Thermofisher Maxima H Minus First Strand cDNA Synthesis according to manufacturer’s instructions. Primers were designed using the Ensembl genome browser, and Roche website. For amplification of human NRP2 (forward primer: 5′-CGGCTTTTGCAGTGGACATC-3′, reverse primer 5′-TGCTCCAGTCCACCTCGTAT-3′) and human Actin (forward primer: 5′-CCAACCGCGAGAAGATGA-3′, reverse primer 5′-CCAGAGGCGTACAGGGATAG-3′). Actin gene expression was used for cDNA normalization.

### Statistical analysis

Comparison between two groups was performed by a Two-tailed unpaired T-test analysis. Difference in bone volume and histomorphometry were assessed by the non-parametric Two-tailed Mann-Whitney Wilcoxon’s test (Graphpad Prism, version 7). A p-value value of 0.05 or below was considered statistically significant.

## Electronic supplementary material

SI Dataset
