# Supplementary material for: Bidirectional regulation of bone formation by exogenous and osteosarcoma-derived Sema3A
Source: Sci Rep. 2018 May 2;8:6877. doi: 10.1038/s41598-018-25290-2 (PMC5932056; doi:10.1038/s41598-018-25290-2)
Supplement: Supplementary file 1 — SI Dataset [file 41598_2018_25290_MOESM1_ESM.doc]

**Bidirectional regulation of bone formation by exogenous and osteosarcoma-derived Sema3A**

Daniëlle de Ridder^1^, Silvia Marino^1^, Ryan T. Bishop^1^, Nathalie Renema^2^, Chantal Chenu^4^, Dominique Heymann^1,3^ and Aymen I. Idris^1^

**Supplementary figures**

**Figure S1.**

**
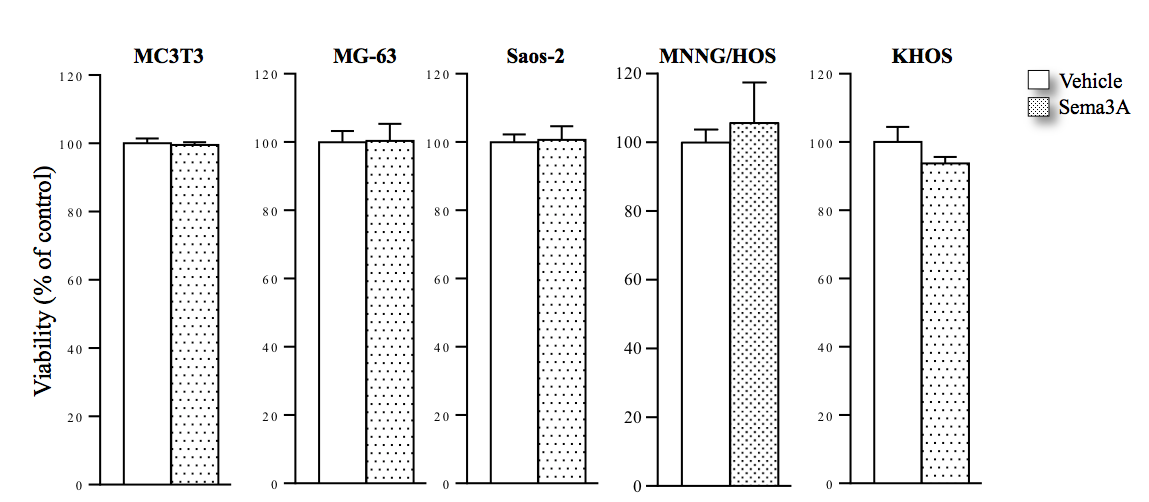
**

**Figure S1. A.** Quantification of MC3T3, MG-63, Saos-2, MNNG/HOS and KHOS cell viability after 48 hour exposure to vehicle (PBS) or Sema3A (300ng/ml). Values are mean ±SEM, n = 3.

**Figure S2.**

**
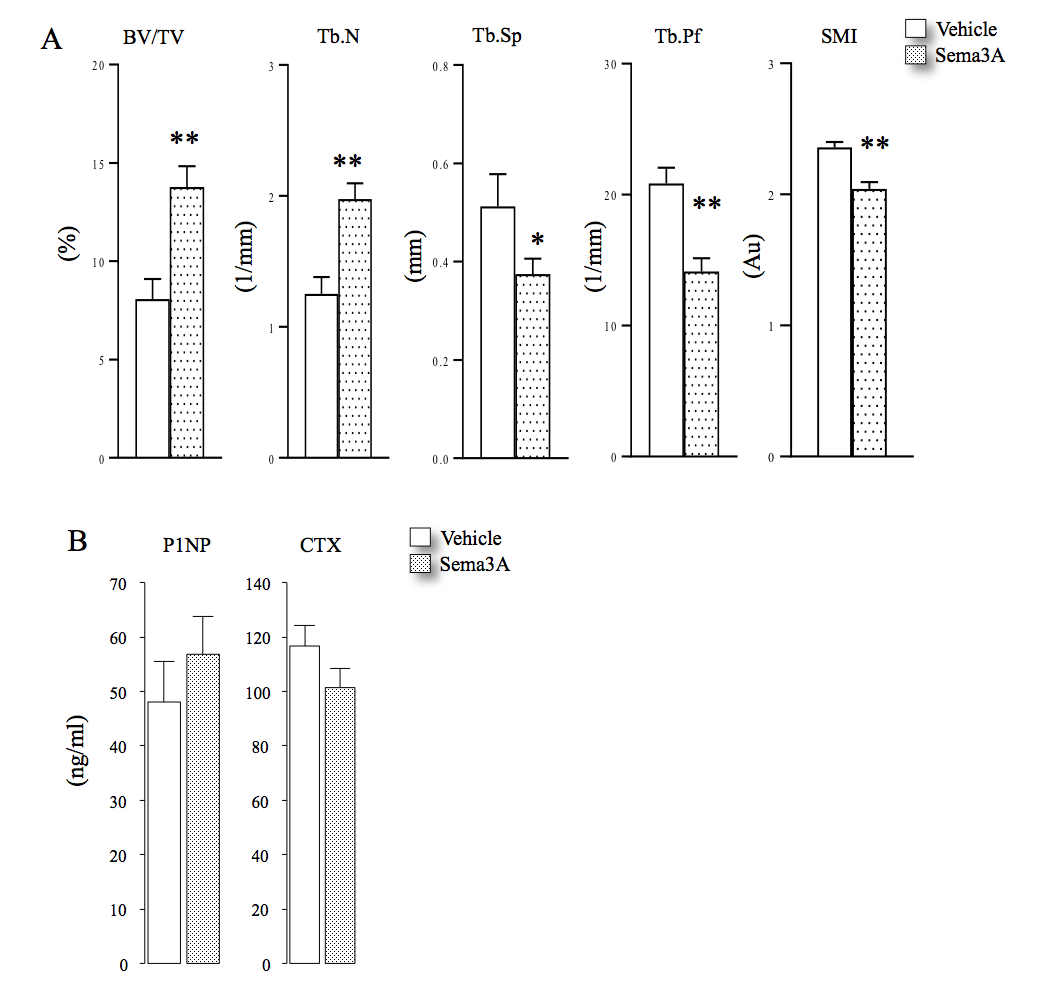
**

**Figure S2 (related to figure 1). A.** Bone parameters from non-inoculated femur of mice inoculated with human KHOS cells and treated with Sema3A (0.7mg/kg) or vehicle (n = 7, 21 days). **B.** Serum level of the bone formation marker N-terminal propeptide of type 1 procollagen (P1NP, left) and bone resorption marker C-terminal telopeptide crosslinks (CTX, right). Values are mean ± SEM; * p < 0.05 and ** p < 0.01.

**Figure S3.**

**
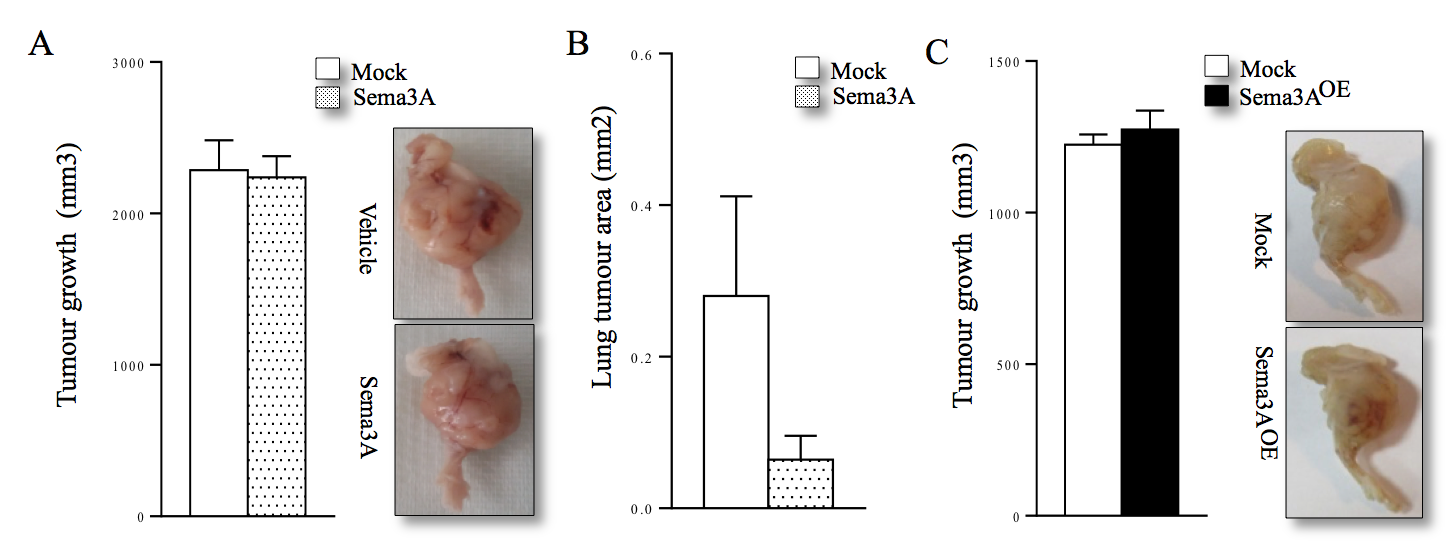
**

**Figure. S3 (related to figures 1 and 4).** *In vivo* tumour growth (**A**) and lung metastasis (**B**) from mice inoculated with human KHOS cells and treated with Sema3A (0.7mg/kg) or vehicle (n = 7) for 21 days. Representative images of tumours from the experiment described are shown in panel A, right. (**C**) *In vivo* tumour growth from mice inoculated with human KHOS cells overexpressing Sema3A (KHOS^OE^, left) or control (mock) (n=10, 16 days). Representative images of tumours from the experiment described are shown in panel C, right. Values are mean ± SEM

**Figure S4.**

**
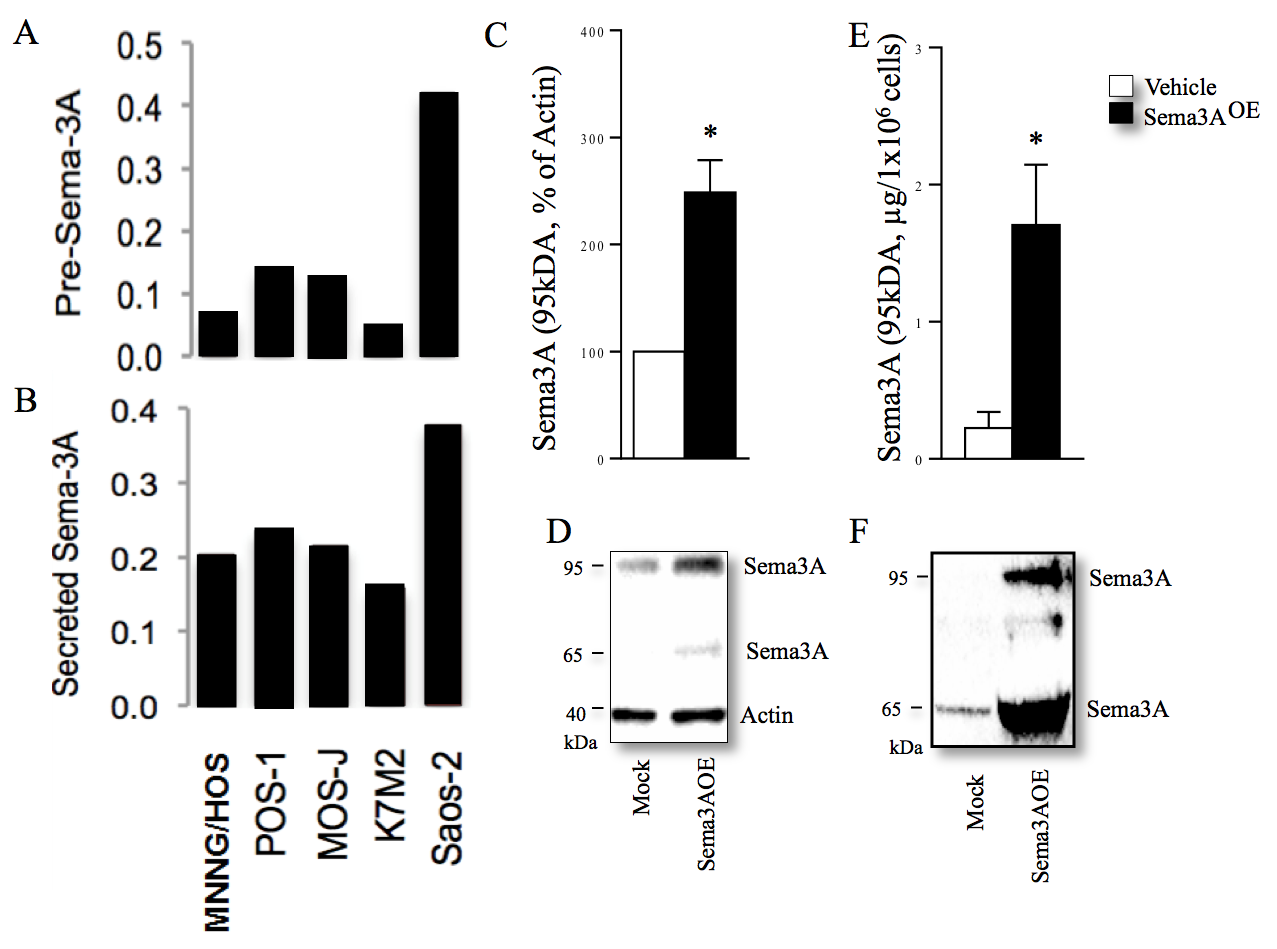
**

**Figure S4 (related to figure 2 and 5).** Expression of secreted Sema3A (**A**) and pre-Sema3A (**B**) in human and murine osteosarcoma cells as assessed by Western blot. (**C, D**) Sema3A expression in human osteosarcoma KHOS cells. (**C)** Differential expression of Sema3A (95kD) in human KHOS cells overexpressing Sema3A (KHOS^OE^, left) and control (mock). (**D**) Representative photomicrographs of Western blot from the experiment described in panel C. (**E**) Protein quantification of Sema3A (95kD) in conditioned medium from human KHOS cells overexpressing Sema3A (KHOS^OE^, left) and control (mock). (**F**) Representative photomicrographs of Western blot from the experiment described in panel E. N = 3 Values are mean ± SEM; * p < 0.05

**Figure S5.**

**
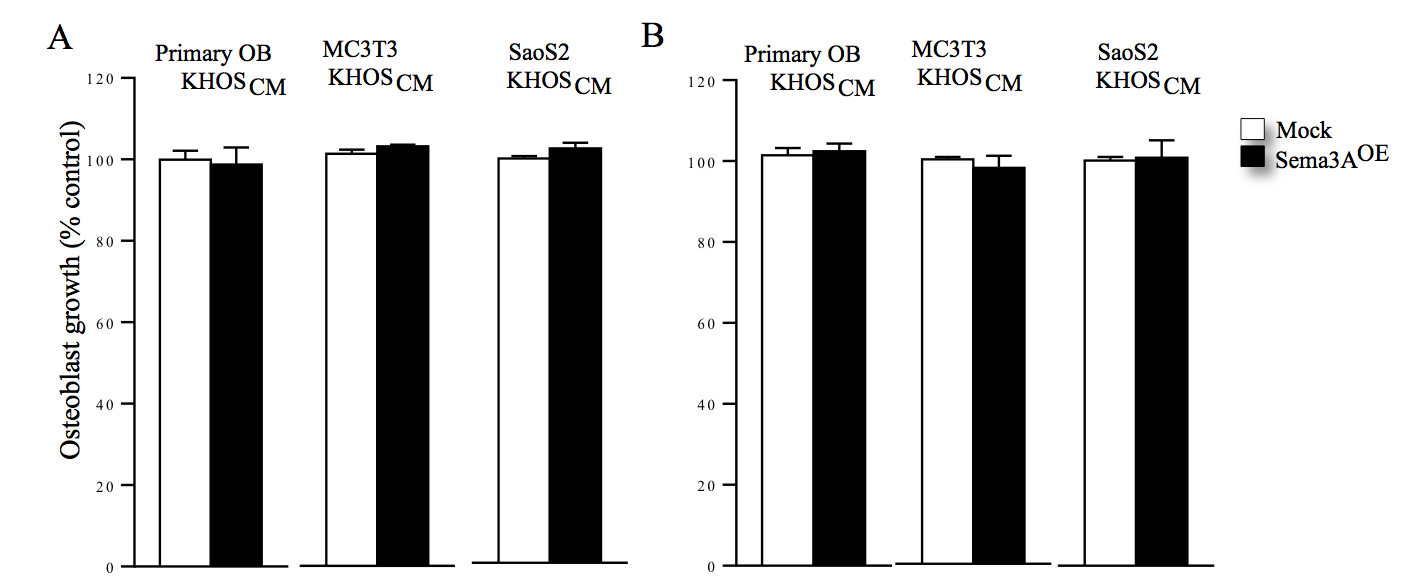
**

**Figure S5 (related to Figure 5).** *In vitro* growth of calvarial osteoblasts, MC3T3 and Saos2 exposed to conditioned medium from KHOS mock and KHOS cells overexpressing Sema3A after 48 hours (**A**) and up to 28 days (**B**) as assessed by AlamarBlue assay. N = 3 Values are mean ± SEM.

**Figure S6.**

**
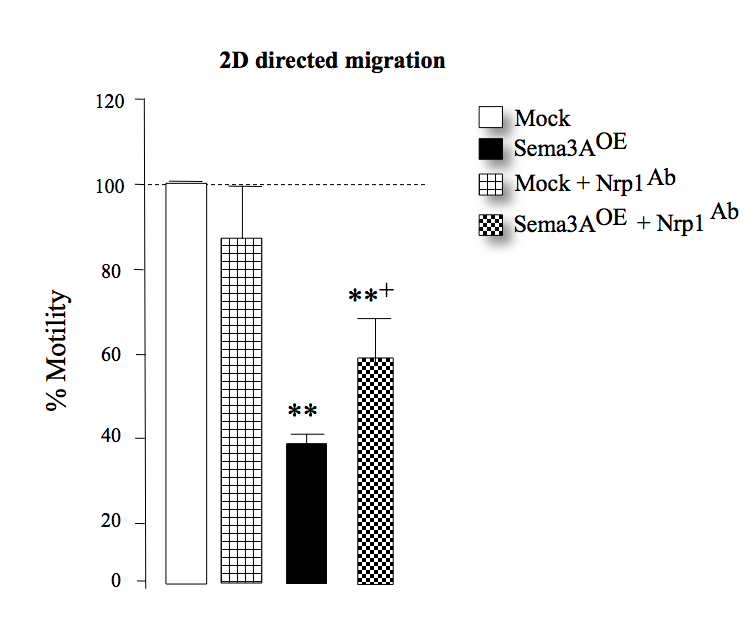
**

**Figure S6 (related to discussion). Nrp1 blockage partially rescues the ability of osteosarcoma-derived Sema3A to reduce KHOS motility.** *In vitro* 2D directed cell migration of human KHOS cells overexpressing Sema3A (Sema3A^OE^) or mock control in the presence and absence of Nrp1 antibody (100ng/ml) after 8 hours as assessed by wound healing. Data are mean ±SD, n = 3 **, p < 0.01 from mock control and +, p < 0.05 from Sema3A^OE^.
